# Performance of A-DROP, NEWS2, and REMS in predicting in-hospital mortality and mechanical ventilation in pneumonia patients in the emergency department: a retrospective cohort study

**DOI:** 10.1186/s12245-024-00792-1

**Published:** 2024-12-27

**Authors:** Netiporn Thirawattanasoot, Brandon Chongthanadon, Onlak Ruangsomboon

**Affiliations:** 1 Department of Emergency Medicine, Faculty of Medicine, Siriraj Hospital, Mahidol University, 2 Wanglang Road, Bangkoknoi, Bangkok, 10700 Thailand; 2 Faculty of Medicine, Siriraj Hospital, Mahidol University, Bangkok, Thailand

**Keywords:** Pneumonia, Early warning score, Emergency department

## Abstract

**Background:**

Pneumonia is a potentially life-threatening respiratory tract infection. Many Early Warning Scores (EWS) were developed to detect patients with high risk for adverse clinical outcomes, but few have explored the utility of these EWS for pneumonia patients in the Emergency Department (ED) setting. We aimed to compare the prognostic utility of A-DROP, NEWS2, and REMS in predicting in-hospital mortality and the requirement for mechanical ventilation among ED patients with pneumonia.

**Methods:**

A retrospective study was conducted at the ED of Siriraj Hospital, Thailand. Adult patients diagnosed with non-COVID-19 pneumonia between June 1, 2021, and May 31, 2022, were included. We calculated and analyzed their EWS at ED arrival. The primary outcome was all-cause in-hospital mortality. The secondary outcome was mechanical ventilation.

**Results:**

We enrolled 735 patients; 272 (37%) died at hospital discharge, and 75 (10.2%) required mechanical ventilation. A-DROP had the highest discrimination capacity for in-hospital mortality (AUROC: 0.698, 95% CI 0.659–0.737) compared to NEWS2 (AUROC 0.657; 95%CI 0.617, 0.698) and REMS (AUROC 0.637; 95%CI 0.596, 0.678). A-DROP also had superior performances than NEWS2 and REMS in terms of calibration, overall model performance, and balanced diagnostic accuracy indices at its optimal cut point (A-DROP *≥* 2). No EWS could perform well in predicting mechanical ventilation.

**Conclusion:**

A-DROP had the highest prognostic utility for predicting in-hospital mortality in non-COVID-19 pneumonia patients in the ED compared to NEWS2 and REMS.

**Supplementary Information:**

The online version contains supplementary material available at 10.1186/s12245-024-00792-1.

## Introduction

Pneumonia, a respiratory tract infection caused by various types of organisms, is one of the leading causes of morbidity and mortality in adult patients. In the United States, the reported annual incidence rate of pneumonia was 24.8 per 10,000 adults in 2015 [1]. In Thailand, the incidence rate was similar at 29.5 per 10,000 adults in 2020 [2]. Regardless of where and when the incidence was reported, these rates tended to increase with advancing age [3–5]. Patients with pneumonia can have a variety of symptoms ranging from mild respiratory distress to acute respiratory failure requiring invasive ventilatory support [5–7]. Effective and timely management is of paramount importance in preventing adverse clinical outcomes, such as mortality and the need for invasive mechanical ventilation. Early detection of patients at high risk of these adverse consequences, especially early in the Emergency Department (ED), is essential for successful management. Accurate risk stratification can help guide treatment and disposition decision-making, thus optimizing patient care. It also helps to allocate hospital resources efficiently [7–9]. 

Many Early Warning Scores (EWS) have emerged as valuable tools that can early recognize deteriorating pneumonia patients in the ED, such as CURB-65 (combination of confusion, urea, respiratory rate, blood pressure, and age) [10], its modified version or A-DROP (age, dehydration, respiratory failure, orientation disturbance, and low blood pressure) [11], and National Early Warning Score 2 (NEWS2). (12–13) These scores, often calculated using a combination of vital signs and clinical parameters, offer a systematic and objective approach to assessing a patient’s condition and predicting the likelihood of clinical deterioration [10–15]. The Rapid Emergency Medicine Score (REMS) is another EWS that has characteristics and objectives similar to others and has proven to accurately prognosticate outcomes of patients with sepsis and COVID-19 pneumonia in the ED. (16–17) However, its prognostic ability for non-COVID pneumonia has never been studied, particularly in the ED setting. The objective of our study was to validate and compare the prognostic utility of A-DROP, NEWS2, and REMS in predicting in-hospital mortality and the requirement for mechanical ventilation among ED patients diagnosed with pneumonia.

## Methods and analysis

### Study Design and setting

This retrospective cohort study was conducted at the ED of Siriraj Hospital, Mahidol University, Bangkok, Thailand. The hospital is a large tertiary-care center with over 2000 hospital beds and an annual number of approximately 1000 ED visits caused by pneumonia. This study was approved by Siriraj Institutional Review Board (certificate of approval: Si557/2022). As the study was retrospective in nature, obtaining informed consent was exempted. This patient cohort has previously been investigated for factors associated with in-hospital mortality comparing between elderly and non-elderly patients, and that study has been published [18]. 

### Participants

Eligible participants were patients 18 years of age or older diagnosed with pneumonia who visited the ED at Siriraj Hospital between June 1, 2021 and May 31, 2022. Pneumonia was defined according to the International Classification of Disease (ICD)-10th version. These diagnoses were manually reviewed by the study investigators (N.T. and B.C.) to confirm patients’ eligibility. Patients with a diagnosis of COVID-19 pneumonia or individuals with conditions other than pneumonia were excluded from the study.

### Data variable and collection

We collected the following patients’ data from their medical records: age, gender, initial vital signs, coexisting medical conditions, mental status upon ED arrival, initial laboratory findings, type of pneumonia diagnosed at the ED, initial oxygen support type, ongoing ventilation support during the hospital stay, ED and hospital disposition, and duration of stay in both the ED and the hospital. All data were extracted by a trained chart abstractor (B.C.) and then reviewed for completeness and correctness by another abstractor (N.T.) using a piloted record form.

### Scoring systems

NEWS2, REMS, and A-DROP are clinical scoring systems with weighted components. NEWS2 is a 0–20 scale with the following parameters: pulse rate, respiratory rate, body temperature, systolic blood pressure, pulse oximetry, and oxygen supplement. REMS assesses patients’ pulse rate, respiratory rate, mean arterial pressure, mental status, pulse oximetry, and age on a 0–26 scale. A-DROP, on a 0–5 scale, considers patients’ age, dehydration status, respiratory failure, orientation, and blood pressure. Table S1 provides further details and the calculation of these three EWS.

### Study objectives and outcomes

The primary objective was to compare the performance of the three EWS, A-DROP, NEWS2, and REMS, in predicting in-hospital mortality in pneumonia patients in the ED. The primary clinical outcome was thus in-hospital mortality. The secondary outcome was invasive mechanical ventilation. We could obtain these outcomes from all patients, and there was no patients discharged against medical advice in the study cohort.

### Statistical analysis

We reported categorical variables using frequencies and percentages and compared them using either the chi-squared test or Fisher’s exact test as appropriate. Continuous variables were reported as mean with standard deviation or median with interquartile range and compared using Student’s t-test or the Mann-Whitney U test for normally distributed and non-normally distributed data, respectively.

We employed various statistics to evaluate the prognostic utility of A-DROP, NEWS2, and REMS for both primary and secondary outcomes. Discrimination was assessed by the area under the receiver operator characteristics curve (AUROC) with its 95% confidence interval (CI). We then compared these AUROCs among the three EWS for each study outcome. The p-values for the pairwise comparison were adjusted for multiple comparisons with Bonferroni correction. Calibration was assessed with calibration plots and the Hosmer-Lemeshow test. Additionally, we employed Nagelkerke’s R-squared to estimate the overall performance of the model.

We further evaluated the clinical utility of all EWS using their optimal cutoff values based on the Youden index. We calculated and reported their sensitivity, specificity, positive likelihood ratio (LR+), negative likelihood ratio (LR-), negative predictive value (NPV), and positive predictive value (PPV) with 95%CIs.

A p-value of 0.05 was considered statistically significant unless specified otherwise. All analyses were performed using SPSS 18.0 (IBM Corp., Chicago, IL), R software version 3.6.1 (R Foundation for Statistical Computing, Vienna, Austria) with the rms, Hmisc, foreign, pROC, sciplot, and dca packages, and MedCalc for Windows version 19 (MedCalc statistical software, Mariakerke, Belgium).

## Results

### Study population

During a one-year period from June 1, 2021, to May 31, 2022, a total of 842 patients with clinical symptoms suggestive of pneumonia visited the ED of Siriraj Hospital. Among these, 735 patients were diagnosed with non-COVID pneumonia and were thus included in the study. No patients had missing EWS values or missing outcomes.

Baseline characteristics of the study population by in-hospital mortality status are presented in Table 1. Patients who died at hospital discharge were significantly older than those discharged alive (*p* = 0.001). There were no significant differences between the two groups regarding underlying conditions, except for malignancy, which was more predominant in patients with in-hospital mortality (*p* < 0.001). Also, they had a higher average Charlson’s comorbidity index (*p* < 0.001). As for initial vital signs, those with in-hospital mortality had significantly lower body temperature, blood pressure, pulse oximetry, and Glasgow Coma Scale (GCS) score despite comparable respiratory and pulse rates compared to patients discharged alive (Table 1). Patients discharged dead also required higher intensity of oxygen supplementation at arrival and had generally worse laboratory results than those discharged alive (Table 1).

**Table 1:** Baseline characteristics of emergency patients with pneumonia

Characteristic	Dead (*n* = 272)		Alive (*n* = 463)		*p*-value	
Age (years)	73.6 *±* 14.1		69.8 *±* 15.3		0.001	
Sex (male)	150 (55.1)		263 (56.8)		0.662	
Underlying disease						
Diabetes mellitus	89 (32.7)		149 (31.7)		0.785	
Coronary artery disease	35 (12.9)		66 (14.3)		0.598	
Cerebrovascular disease	75 (27.6)		108 (23.3)		0.199	
CKD stage 3–5 or ESRD	54 (19.9)		92 (19.9)		0.995	
Chronic lung disease	61 (22.4)		115 (24.8)		0.459	
Malignancy	115 (42.3)		132 (28.5)		< 0.001	
Charlson’s comorbidity index	6.5 *±* 2.6		5.5 *±* 2.8		< 0.001	
Vital signs and mental status at ED arrival						
Body temperature (^o^C)	36.8, 0.8		37.0, 1.2		0.003	
Respiratory rate (breaths/min)	33.0 *±* 8.3		32.6 *±* 8.9		0.525	
Pulse rate (beats/min)	104.6 *±* 25.9		102.9 *±* 23.9		0.381	
Systolic blood pressure (mmHg)	126.2 *±* 35.7		138.1 *±* 34.2		< 0.001	
Diastolic blood pressure (mmHg)	73.4 *±* 21.1		78.7 *±* 19.8		0.001	
Oxygen saturation (%)	88, 13.8		93, 10		< 0.001	
Glasgow coma scale score	12, 6		15, 3		< 0.001	
Early warning scores at ED arrival						
A-DROP	2.4 *±* 1.1		1.6 *±* 1.1		< 0.001	
NEWS2	9.3 *±* 2.8		7.7 *±* 2.8		< 0.001	
REMS	10.8 *±* 3.3		9.2 *±* 3.2		< 0.001	
Initial oxygen support type						
None	24 (8.8)		65 (14.0)		< 0.001	
Cannula	86 (31.6)		183 (39.5)			
Non-rebreather mask	120 (44.1)		122 (26.3)			
NIV or HFNC	13 (4.8)		47 (10.2)			
Endotracheal intubation	29 (10.7)		46 (9.9)			
Initial laboratory results						
Hemoglobin (g/dL)	9.9 *±* 2.6		10.8 *±* 2.6		< 0.001	
White blood cells (x1000 count/µL)	11.8, 8.5		11.1, 7.8		0.599	
Neutrophil (%)	83.0, 17.8		81.4, 15.2		0.065	
Platelet (x1000 count/µL)	248.9 *±* 147.0		269.8 *±* 141.3		0.057	
GFR (mL/min/1.73mm2)	66.1 *±* 37.2		72.3 *±* 38.0		0.031	
Type of pneumonia						
Community-acquired pneumonia	144 (52.9)		293 (63.3)		0.020	
Hospital-acquired or healthcare-associated pneumonia	124 (45.6)		166 (35.9)			
Ventilator-associated pneumonia	4 (1.5)		4 (0.9)			
Length of stay						
ED stay (hours)	17, 28.8		12, 18		< 0.001	
Hospital stay (days)	3, 7		6, 12		0.006	

Note: data presented as n (%), mean *±* SD or median, interquartile range

Abbreviations: CKD, chronic kidney disease; ESRD, end-stage renal disease; ED, emergency department; NEWS2, National Early Warning Score 2; REMS, Rapid Emergency Medicine Score; NIV, non-invasive ventilation; HFNC, high-flow nasal cannula; GFR, glomerular filtration rate

### Scoring system

**Fig. 1:**
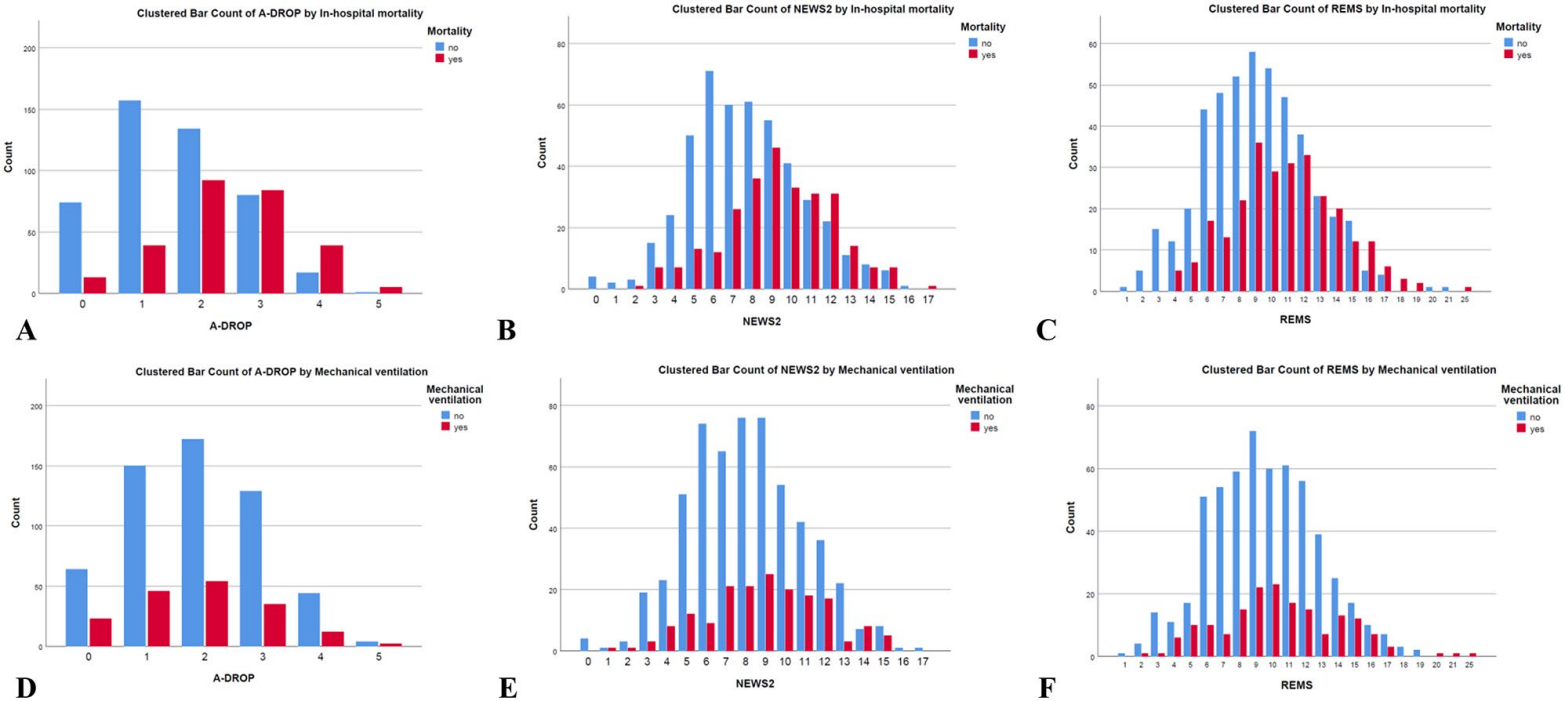
Distribution of early warning scores and study outcomes stratified by each early warning score in emergency patients with pneumonia. For in-hospital mortality: (**A**) A-DROP score, (**B**) NEWS2 score, (**C**) REMS score. For mechanical ventilation: (**D**) A-DROP score, (**E**) NEWS2 score, (**F**) REMS score. Abbreviations: NEWS2, National Early Warning Score 2; REMS, Rapid Emergency Medicine Score

All EWS were significantly higher on average among patients who died at discharge compared to those discharged alive (all *p* < 0.001) (Table 1). The distribution of EWS scores across the patient cohort is depicted in Fig. 1. Notably, a substantial proportion of patients with higher EWS scores experienced the primary outcome, suggesting a robust and positive correlation between EWS values and in-hospital mortality. However, this finding was not as prominent for mechanical ventilation.

### Score performance

**Fig. 2:**
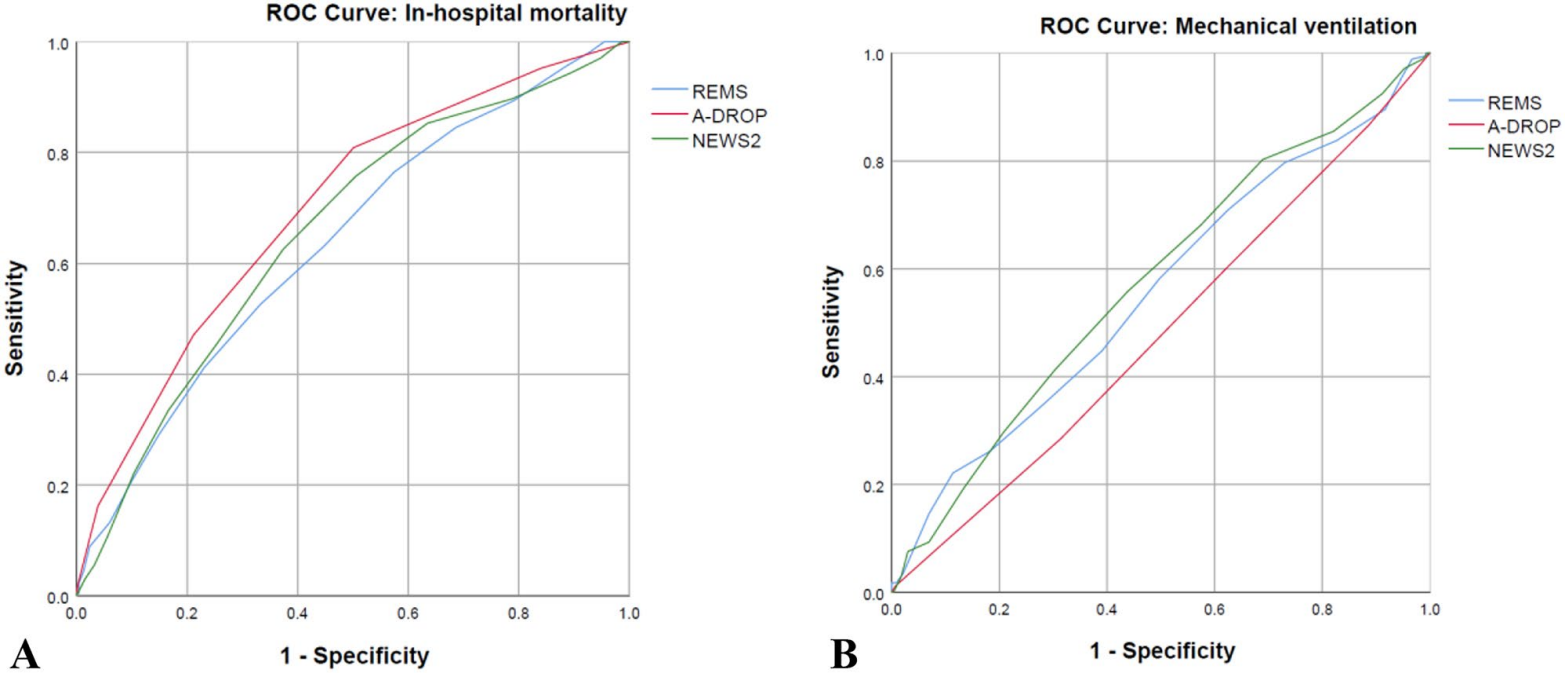
Receiver operator characteristic curves for early warning scores for in-hospital mortality and mechanical ventilation in emergency patients with pneumonia. (**A**) In-hospital mortality. (**B**) Mechanical ventilation. Abbreviations: NEWS2, National Early Warning Score 2; REMS, Rapid Emergency Medicine Score

Discrimination assessed with AUROC for in-hospital mortality showed that all three EWS were close in performance but A-DROP was the best (AUROC 0.698; 95%CI 0.659, 0.737), followed by NEWS2 (AUROC 0.657; 95%CI 0.617, 0.698) and REMS (AUROC 0.637; 95%CI 0.596, 0.678) (Table 2),and the difference between A-DROP was only statistically significant compared to REMS but not NEWS2 (Table 3). All three EWS did not perform well in predicting mechanical ventilation, with the discrimination capacity not exceeding 0.6 and A-DROP having an AUROC below 0.5 (Table 2; Fig. 2).

**Table 2:** Early warning score performance and clinical utility for in-hospital mortality and mechanical ventilation in emergency patients with pneumonia

	Discrimination	Calibration	Overall performance	Clinical utility						
Score	AUROC (95%CI)	Hosmer-Lemeshow Test	Nagelkerke’s *R*-Square (%)	Score category	Sensitivity (95%CI)	Specificity (95%CI)	PPV (95%CI)	NPV (95%CI)	LR+ (95%CI)	LR- (95%CI)
In-hospital mortality										
A-DROP	0.698 (0.659, 0.737)	0.391	15.7	A-DROP *≥* 2	80.9 (75.7–85.4)	49.9 (45.2–54.5)	48.7 (44.0-53.4)	81.6 (76.6–86.0)	1.6 (1.5–1.8)	0.4 (0.3–0.5)
NEWS2	0.657 (0.617, 0.698)	0.160	8.6	NEWS2 *≥* 8	75.7 (70.2–80.7)	49.5 (44.8–54.1)	46.8 (42.1–51.6)	77.6 (72.4–82.3)	1.5 (1.3–1.7)	0.5 (0.4–0.6)
REMS	0.637 (0.596, 0.678)	0.922	7.6	REMS *≥* 11	52.6 (46.5–58.6)	66.7 (62.6–71.0)	48.1 (42.3–54.0)	70.5 (66.0-74.8)	1.6 (1.3–1.9)	0.7 (0.6–0.8)
**Mechanical ventilation**										
A-DROP	0.482 (0.432, 0.531)	0.954	0.1	A-DROP *≥* 5	1.2 (0.1–4.1)	99.3 (98.2–99.8)	33.3 (4.3–77.7)	76.7 (73.4–79.7)	1.6 (0.3–8.9)	1.0 (1.0–1.0)
NEWS2	0.576 (0.527, 0.625)	0.541	1.8	NEWS2 *≥* 9	55.8 (48.1–63.4)	56.1 (51.9–60.3)	28.0 (23.3–33.1)	80.6 (76.3–84.4)	1.3 (1.1–1.5)	0.8 (0.7-1.0)
REMS	0.557 (0.506, 0.607)	0.120	1.4	REMS *≥* 14	22.1 (16.1–29.0)	88.6 (85.7–91.1)	37.3 (27.9–47.4)	78.8 (75.4–82.0)	1.9 (1.4–2.8)	0.9 (0.8-1.0)

Notes: cut-off values for all early warning scores were chosen by optimal Youden Index

Abbreviations: AUROC, area under the receiver operator characteristics curve; CI, confidence interval; LR+, positive likelihood ratio; LR-, negative likelihood ratio; NPV, negative predictive value; PPV, positive predictive value; NEWS2, National Early Warning Score 2; REMS, Rapid Emergency Medicine Score

**Table 3:** Pairwise comparisons of area under the receiver operator characteristic curve of early warning scores for in-hospital mortality and mechanical ventilation among emergency patients with pneumonia

		In-hospital mortality		
		A-DROP	NEWS2	REMS
**Mechanical ventilation**	**A-DROP**		0.151	0.004
	**NEWS2**	0.002		0.949
	**REMS**	0.003	0.999	

Note: p-value for overall difference among all scores = 0.005 for in-hospital mortality and < 0.001 for mechanical ventilation. The p-values reported were already adjusted for multiple comparisons with Bonferroni correction

Abbreviations: NEWS2, National Early Warning Score 2; REMS, Rapid Emergency Medicine Score

**Fig. 3:**
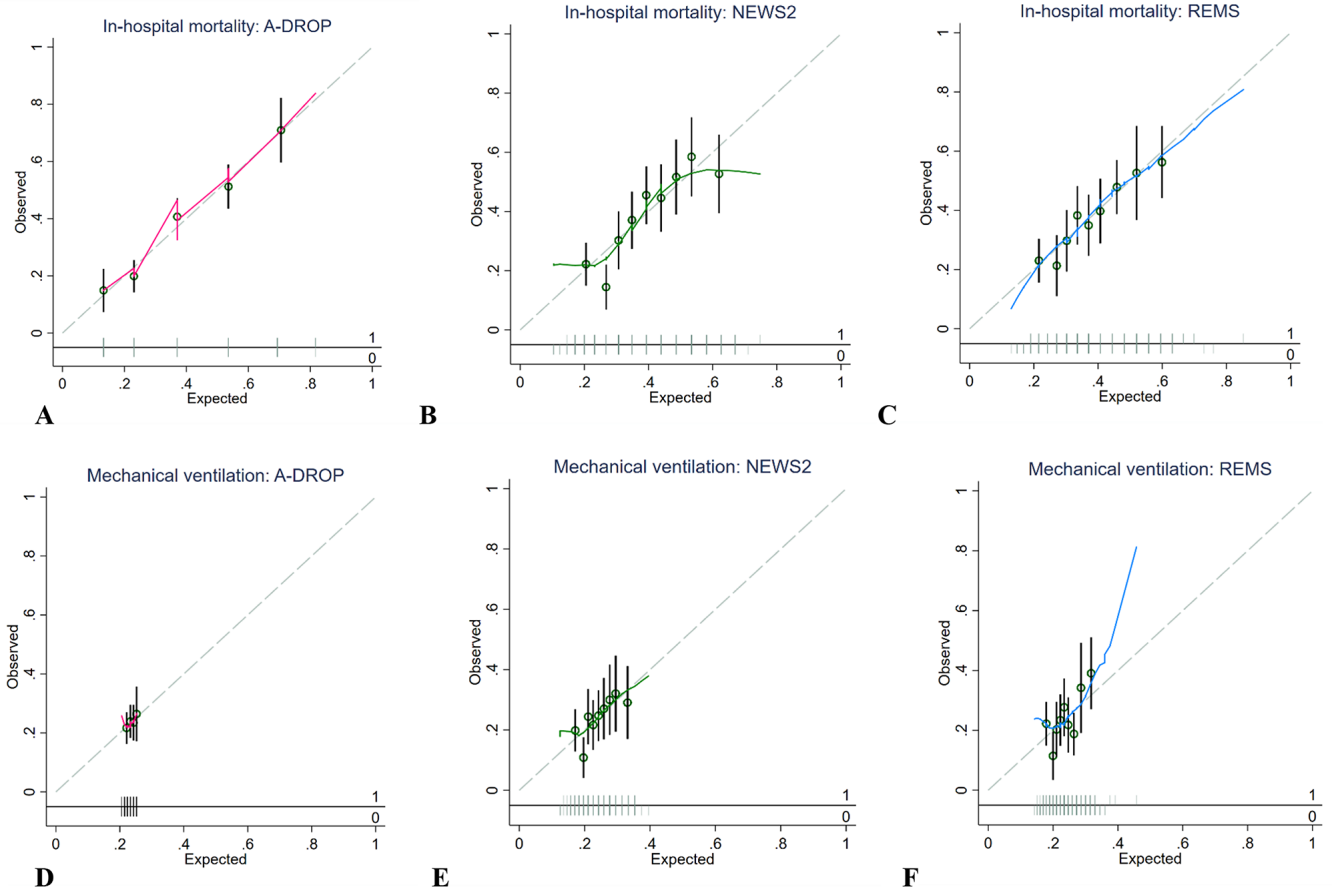
Calibration plots of early warning scores in emergency patients with pneumonia. For in-hospital mortality: (**A**) A-DROP score, (**B**) NEWS2 score, (**C**) REMS score. For mechanical ventilation: (**D**) A-DROP score, (**E**) NEWS2 score, (**F**) REMS score. Hollow circles denote groups of predicted risk. Vertical line through hollow circles denote 95% confidence intervals. The distribution of non-events of the outcome (0) and events of the outcome (1) by expected probability are denoted by the rug plot (light grey) along the x axis. Abbreviations: NEWS2, National Early Warning Score 2; REMS, Rapid Emergency Medicine Score

A-DROP and REMS calibrated well along different predictive probabilities of in-hospital mortality, as shown in Fig. 3 and confirmed by the Hosmer-Lemeshow test in Table 2. In contrast, assessing calibration for mechanical ventilation was limited due to only patients with lower risks being represented in the study population (Fig. 3).

The overall performance, evaluated using Nagelkerke’s R-Square, found A-DROP to have the best performance for in-hospital mortality, in concordance with its superior discrimination and calibration capacity (Table 2). Regardless, no EWS showed favourable overall performance for mechanical ventilation.

Table 2 also depicts the clinical utility of EWS. The optimal cut points for in-hospital mortality based on the Youden index were A-DROP *≥* 2, NEWS2 *≥* 8 and REMS *≥* 11, while the optimal cut points for mechanical ventilator were A-DROP *≥* 5, NEWS2 *≥* 9 and REMS *≥* 14. For in-hospital mortality, A-DROP *≥* 2 had the most balanced sensitivity and specificity, highest PPV, NPV, LR + and lowest LR- (sensitivity 80.9; 95% CI 75.7–85.4, specificity 49.9; 95% CI 45.2–54.5, PPV 48.7; 95% CI 44.0-53.4, NPV 81.6; 95% CI 76.6–86.0, LR + 1.6; 95% CI 1.5–1.8, LR- 0.4; 95% CI 0.3–0.5). A-DROP could also detect the highest number of patients with in-hospital mortality, with the highest proportion of true positives and the lowest proportion of false positives (Table 4). For mechanical ventilation, although NEWS2 had the most balanced sensitivity and specificity, both were lower than 60% (Table 2), and it yielded the lowest proportion of true positives (Table 4).

**Table 4:** Classification according to early warning scores

Outcomes	All patients, no (%)	A-DROP, n (%)		NEWS2, n (%)		REMS, n (%)	
		< 2 (*n* = 283)	*≥* 2 (*n* = 452)	< 8 (*n* = 295)	*≥* 8 (*n* = 440)	< 11 (*n* = 438)	*≥* 11 (*n* = 297)
In-hospital death	272 (37.0)	52 (18.4)	220 (48.7)	66 (22.4)	206 (46.8)	129 (29.5)	143 (48.1)
**Outcomes**	**All patients, no (%)**	**A-DROP, n (%)**		**NEWS2, n (%)**		**REMS, n (%)**	
		**< 5 **(***n* = 729)**	**≥ 5** (***n* = 6)**	**< 9 **(***n* = 392)**	**≥ 9 **(***n* = 343)**	**< 14 **(*n = 633)*	**≥ 14** (***n = 102)***
Mechanical ventilation	172 (23.4)	170 (23.3)	2 (33.3)	76 (19.4)	96 (28.0)	134 (21.2)	38 (37.3)

Abbreviations: NEWS2, National Early Warning Score 2; REMS, Rapid Emergency Medicine Score

## Discussion

This single-center retrospective study validated and compared A-DROP, NEWS2, and REMS in predicting adverse clinical outcomes of patients diagnosed with non-COVID-19 pneumonia in an ED setting. We found that A-DROP outperformed REMS and NEWS2 in predicting in-hospital mortality across a wide range of statistics employed. A-DROP demonstrated the highest AUROC that calibrated well across various predictive probabilities, the best overall performance, and superior prognostic accuracy compared to NEWS2 and REMS. However, no score could perform well in predicting the need for mechanical ventilation, with all EWS having less than optimal indices for almost all metrics analyzed.

Diagnosing pneumonia and assessing the severity of patients is crucial in the ED because it helps to closely monitor and decide on appropriate disposition for high-risk patients, thus potentially preventing adverse consequences. We found that A-DROP could effectively and accurately predict in-hospital mortality, superior to NEWS2 and REMS, even in the ED setting. Although previous evidence has shown that A-DROP is an EWS that has clinical utility in prognosticating poor outcomes in inpatient settings [11, 19, 20], limited studies have validated its utility for the same purposes in the ED. A previous study has explored and reported that A-DROP can accurately help identify low-risk pneumonia patients for safe discharge in the ED [21]. Regardless, no previous studies have validated or compared A-DROP with other EWS in stratifying high-risk patients in the ED. Therefore, we have added to the body of evidence that the utility of A-DROP expands beyond inpatient settings. The reason for its superiority over NEWS2 and REMS, the other more ED-specific scores, might have lied within their components. Pulse rate is one of the factors used in calculating REMS and NEWS2, but not in A-DROP. Our previous study of the same patient cohort showed that pulse rate was not associated with in-hospital mortality [18], as it could have been affected by many other reasons not specific to pneumonia, for example, agitation and pain. Therefore, EWS consisting of this component may not perform well compared to A-DROP, whose components are more specific to pneumonia, as it was the derivative of CURB-65, a score specifically invented to differentiate pneumonia patients.

In the present study, we found NEWS2 and REMS to have comparable prognostic utility. This finding was similar to other previous studies performed in the ED, in which NEWS2 and REMS had favourable and relatively comparable performance, whether they were assessed in general ED patients [22], patients with sepsis [16], or COVID-19 pneumonia patients [17]. It was important to note that A-DROP was not evaluated in these previous studies. This suggests future areas of research, especially for COVID-19 pneumonia, as A-DROP may also has favourable and superior prognostic ability to the other ED-based EWS in these patient populations.

Regardless, it is essential to mention that the AUROC for A-DROP was not very high. Still, it was comparable to that of a study including patients diagnosed with aspiration pneumonia [19], but slightly lower than that reported in another study involving hospitalized patients with community-acquired pneumonia [23]. The discordance was most likely due to different patient population and their characteristics, which emphasizes the need for a validation study, such as ours, before employing EWS for clinical use for any specific patient population.

While A-DROP performed well for in-hospital mortality, it failed to do so for mechanical ventilation. In fact, none of the three EWS had adequate prognostic and clinical utility for this clinical outcome, with varying performances across different metrics evaluated. This could have also been explained by the specificity of the score components towards the outcome. A decision to intubate may come from factors other than the patient’s condition, such as the ability to monitor patients. Also, many of the components of these EWS represent the consequence of end-organ damage, especially those of A-DROP, which may not be directly relevant to mechanical ventilation. Healthcare providers in the ED may make the decision to intubate before patients experience end-organ damage. These reasons could have explained the less-than-optimal predictive performance of the three EWS under study, especially for A-DROP. With regards to NEWS2 and REMS, our findings were discordant with the previous study in COVID-19 pneumonia patients in the ED [17], where REMS and NEWS2 could demonstrate better performance in predicting mechanical ventilation compared to the present study. This discordance could have been because of the different characteristics between COVID-19 and non-COVID pneumonia patients, with COVID-19 patients being younger with fewer comorbidities. Consequently, the decision to intubate COVID-19 patients could have been more straightforward and less conservative compared to the non-COVID population in the present study.

### Limitations

This study had some limitations. First, it was a single-center study in a large tertiary hospital, potentially limiting its generalizability to other settings. Due to the distinct characteristic of our ED, the study population was generally more severe than those in other diagnostic/prognostic studies of dyspneic and pneumonia patients in the ED, with the present study cohort having more severe vital signs and higher mortality and mechanical ventilation rates. (17, 21–22, 24–25) This could affect accuracy indices and emphasizes the need to externally validate these EWS in other different settings. Second, the study was retrospective in nature, which could have suffered from the drawbacks of retrospective studies in general. Third, the primary outcome was all-cause mortality non-specific to pneumonia. The non-specific mortality outcome was chosen because it was challenging to determine disease-specific mortality retrospectively. Also, the outcome was assessed at hospital discharge, which could have also been non-specific to pneumonia. Future multicenter prospective studies evaluating adverse clinical outcomes directly related to pneumonia are required to strengthen our study’s findings.

## Conclusion

A-DROP, a modified version of CURB-65, had better overall performance than NEWS2 and REMS in predicting in-hospital mortality among non-COVID-19 pneumonia patients in the ED. Regardless, none of the three EWS performed well in predicting the need for mechanical ventilation.

## Electronic supplementary material

Below is the link to the electronic supplementary material.

Supplementary Material 1

## Data Availability

The datasets generated and analyzed in this study are not publicly accessible. However, they can be obtained from the corresponding author upon a reasonable request.

## Abbreviations

REMS: The rapid emergency medicine score
NEWS2: National early warning score 2
ED: Emergency department
EWS: Early warning score
COVID-19: Coronavirus disease 2019
ICD: The international classification of disease
GCS: Glasgow coma scale
AUROC: Area under the curve of the receiver operator characteristics curves
LR+: Positive likelihood ratio
LR-: Negative likelihood ratio
NPV: Negative predictive value
PPV: Positive predictive value
CI: Confidence interval
