# Supplementary material for: Performance of A-DROP, NEWS2, and REMS in predicting in-hospital mortality and mechanical ventilation in pneumonia patients in the emergency department: a retrospective cohort study
Source: Int J Emerg Med. 2024 Dec 27;17:198. doi: 10.1186/s12245-024-00792-1 (PMC11674152; doi:10.1186/s12245-024-00792-1)
Supplement: Supplementary file 1 — Supplementary Material 1 [file 12245_2024_792_MOESM1_ESM.pdf]

Table S1. Components and scores of the A-DROP, NEWS2, and REMS

A-DROP (0-5)	NEWS2 (0-20)	REMS (0-26)
Age (0-1)<br>male <70 years or female <75 years (0),<br>male ≥70 years or<br>female ≥75 years (1)	HR (0-3)<br>51-90/min (0),<br>41-50 or 90- 110/min (1),<br>111-130/min (2),<br>≤40 or >131/min (3)	HR (0-4)<br>70-109/min (0),<br>55-69/min or 110-139/min (2),<br>40-54/min or 140-179/min (3),<br>≤ 39/min or ≥ 179/min (4)
Dehydration (0-1)<br>BUN ≤210 mg/L (0),<br>BUN >210 mg/L (1)	RR (0-3)<br>12-20/min (0),<br>9-11/min (1), 21-24/min (2),<br>≤8 or ≥25/min (3)	RR (0-4)<br>12-24/min (0),<br>10-11/min or 25-34/min (1),<br>6-9/min (2),<br>35-49/min (3),<br>≤5/min or >49/min (4)
Respiratory failure (0-1)<br>SaO_2 >90% or<br>PaO_2 >60 mmHg (0),<br>SaO_2 ≤90% or<br>PaO_2 ≤60 mmHg (1)	Body temperature (0-3)<br>36.1-38°c (0),<br>35.1-36 or 38.1-39°c (1), >39.1°c (2),<br>≤35 °c (3)	Age (0-6)<br><45 years (0),<br>45-54 years (2),<br>55-64 years (3),<br>65-74 years (5),<br>>74 years (6)
Orientation disturbance (confusion) (0-1)<br>No (0),<br>Yes (1)	SBP (0-3)<br>111-219 mmHg (0),<br>101-110 mmHg (1),<br>91-100 mmHg (2),<br>≤90 or ≥220 mmHg (3)	MAP (0-4)<br>70-109 mmHg (0),<br>50-69 mmHg or 110-129 mmHg (2),<br>130-159 mmHg (3),<br>≤ 49 mmHg. or >159 mmHg (4)
SBP (0-1)<br>>90 mmHg (0),<br>≤90 mmHg (1)	Neurological (0-3)<br>Alert (0),<br>reacting to voice or reacting to pain or unresponsive (3)	Glasgow coma score (0-4)<br>14 or 15 (0),<br>11-13 (1),<br>8-10 (2),<br>5-7 (3),<br>3 or 4 (4)
	Oxygen saturation (0-3)<br>≥96% (0),<br>94-95% (1),<br>92- 93% (2),<br>≤91% (3)	Oxygen saturation (0-4)<br>>89% (0),<br>86-89% (1),<br>75- 85% (3),<br><75% (4)
	Oxygen supplement (0-2)<br>No (0),<br>Yes (2)	

Abbreviations: BUN, blood urea nitrogen; SaO_2, oxygen saturation of arterial blood; PaO_2, partial pressure of oxygen in the arterial blood; SBP, systolic blood pressure; NEWS, national early warning score; HR, heart rate; RR, respiratory rate; REMS, rapid emergency medicine score; MAP, mean arterial pressure
